# Processing-Induced Inhomogeneity of Yield Stress in Polycarbonate Product and Its Influence on the Impact Behavior

**DOI:** 10.3390/polym8030072

**Published:** 2016-03-04

**Authors:** Yingjie Xu, Huan Lu, Tenglong Gao, Weihong Zhang

**Affiliations:** Engineering Simulation and Aerospace Computing (ESAC), Northwestern Polytechnical University, Xi’an 710072, China; luhuan@mail.nwpu.edu.cn (H.L.); gaotl@mail.nwpu.edu.cn (T.G.)

**Keywords:** polycarbonate, injection molding process, yield stress, inhomogeneity, Izod impact, finite element simulation

## Abstract

In this study, an integrated methodology for impact analysis of polycarbonate (PC) product is proposed which incorporates the processing-induced inhomogeneity of yield stress. A previously developed model is extended to predict the inhomogeneous yield stress distribution along the specimen by using the thermal history experienced during injection molding. A strain rate-dependent elastic-plastic model combining the processing-induced yield stress is applied to model the mechanical behavior of PC. Finite element simulation for notched Izod impact test is then conducted to analyze the impact behaviors of PC specimens with different thermal histories. Numerical results of the fracture energies are compared with experimental measurements.

## 1. Introduction

Polycarbonate (PC) is frequently used in impact protection applications, including aircraft canopies, face shields, goggles, and windshields, windows and blast shields, due to a combination of high transparency and impact resistance. Thus, impact response of the PC product is a subject of critical interest. For a correct and safe PC product design, the understanding of its mechanical performance under impact loading is of utmost importance. In the literature, the impact response of PC has been extensively studied by experimentation or modeling studies. Gas gun impact tests were carried out by Gunnarson, *et al.* [1], Qasim [2], Mckenzie, *et al.* [3], Wright, *et al.* [4], and Li and Goldsmith [5] to investigate the deformation and fracture mechanisms of thin PC plates under high velocity impact. In addition, Izod impact tests were widely used to study the low velocity impact behavior of PC, such as Fraser, *et al.* [6], Cheng, *et al.* [7], Lombardo, *et al.* [8], and Inberg, *et al.* [9]. A number of numerical studies based on the finite element method have been also performed to investigate the dynamic response of PC under impact loading. Representative work includes the following: Dorogoy, *et al.* [10,11] conducted systematic studies on the inclined ballistic impact in thick PC plates by 3D transient non-linear adiabatic finite element simulations. Qasim [12] simulated the plastic deformation of a thin rectangular PC plate under low velocity impact. Bobaru, *et al.* [13] presented computational results for the impact of a spherical projectile on a thin glass plate with a thin PC backing plate. Tvergaard, *et al.* [14] analyzed the evolution of stress and strain fields in PC specimens during the Izod impact test.

PC products are primarily produced by injection molding process [15,16]. In such a process, the melted PC is injected into a mold cavity with a desired shape and then cooled down under a high packing pressure. In the studies by Govaert and co-workers [17,18,19,20,21], it has been proven that the thermal history experienced upon solidification from the melt during the injection molding process has an influence on the yield stress of molded PC products. Furthermore, the thermal history of various positions along a product is inhomogeneous, especially for the product with complex geometry. As a consequence, the yield stress would be different at different locations within a molded PC product [19,20].

Therefore, for a PC product under impact loading, we can conclude that the thermal history during injection molding has inevitable consequences for its impact behavior. However, few experimental or modeling studies have been carried out for investigating the influence of thermal history on the impact behavior of PC. In our previous study [22], we developed an integrated methodology which combines the evaluation of the yield stress of PC after processing and the finite element computation of the impact behavior for simple-shaped PC specimens in a low velocity Izod impact test. In that study, a thickness-weighted average [19] neglecting the inhomogeneous yield stresses distribution was used to simplify the impact simulations. However, for complex-shaped PC products, the difference of thermal history for the material elements at different locations is far larger compared with the simple-shaped products. In that case, the inhomogeneity of yield stress within the products is considerable and must be carefully described in the computation model for accurately predicting the consequence of processing thermal history on the impact behavior.

Thus, the purpose of this paper is to extend our previous model [22] to incorporate the inhomogeneity of yield stresses along the geometrical dimensions of PC products. The inhomogeneous yield stress distribution along the injection molded specimen is calculated by the method developed by Govaert, *et al.* [17,18,19] and used for the notched Izod impact simulations, instead of an average value for the whole specimen. The notched Izod impact tests are carried out for PC specimens with different processing conditions. The numerical results are compared to the test results of PC specimens with different processing conditions. Numerical results of the fracture energies are compared with experimentally measured data to illustrate and assess the predictive capability of the proposed model. The novelty of this work compared with our previous paper [22] is that we are extending our previous work to incorporate the inhomogeneity of yield stresses. An integrative simulation framework to incorporate the inhomogeneous yield stress distribution into the impact simulations is developed. In order to illustrate the advantage of using the inhomogeneity of yield stress, comparisons and discussions between the fracture energy results of averaged yield stress and inhomogeneous yield stress are presented. It is shown that an improved agreement between experimental results and simulations is achieved considering the inhomogeneity of yield stress.

## 2. The Predictive Model of Processing-Induced Yield Stress of PC

The prediction of yield stress of PC from processing thermal history is based on a phenomenological approach developed by Govaert, *et al.* [17,18,19]. It is assumed the physical processes of the evolution of yield stress during processing are identical to those governing the evolution of yield stress during annealing. In previous study [22], we conducted a series of experiments to investigate the evolution of yield stress of PC during annealing process and determine the parameter values used for evaluating the yield stress. Thus, here only a basic introduction is presented. The detailed description of the experiment and the model can be found in our previous study [22].

Firstly, the dog-bone tensile samples of PC Lexan 141R with two different mold temperatures (90 and 140 °C) are injection molded. Then, all the samples are annealed for a specified period of time at four different annealing temperatures (80, 100, 120, and 130 °C). After required annealing period, samples are slowly cooled to room temperature. The yield stresses for all the samples are then determined by tensile test under a constant strain rate of 10^−2^ s^−1^, which are further combined into a single master curve for a reference annealing temperature of 23 °C by using annealing time-temperature superposition [17]. Based on this master curve, the yield stress during the annealing process can be evaluated using a logarithmic evolution equation [17,18,19]:

(1) $$\sigma_{y}\left(t\right)=\sigma_{y,\;0}+\;c\cdot \log\left(t_{eff}+\;t_{a}\right)$$

where $\sigma_{y,\;0}$ is the intersection of the linear relation of yield stress with the logarithm of time and *c* is the slope. The values of $\sigma_{y,\;0}$ and c are directly obtained from the master curves for a strain rate of 10^−2^ s^−1^: $\sigma_{y,\;0}=25.8\;MPa$ and *c* = 3.8 *MPa*. $t_{a}$ is only depended on the processing thermal history. $t_{eff}$ is the effective time accelerated by temperature which captures the thermal history of the material experiences during annealing, T(t), and is defined as:

(2) $$t_{eff}=\int_{0}^{t}a_{T}^{-1}\left(T\left(t\right)\right)dt$$

where $a_{T}\left(T\right)$ is the shift factor used to construct the master curve. It can be described by an Arrhenius relation:

(3) $$a_{T}\left(T\right)=\exp\left(\frac{\Delta U_{a}}{R}\cdot \left(\frac{1}{T}-\frac{1}{T_{ref}}\right)\right)$$

where $\Delta U_{a}$ is the activation energy equaling to 205 kJ/mol [19]. *R* is the universal gas constant, *T* is the annealing temperature, and $T_{ref}$ is the reference temperature.

Assuming the cooling process during injection molding which starts at the moment that the temperature passes through the glass transition temperature could be regarded as an effective annealing process, the thermal history the material receives during cooling process can be determined as:

(4) $$\begin{array}{ll} T>T_{g}: & t_{eff}^{c}=0\\ T\leq T_{g}: & t_{eff}^{c}=\int_{0}^{t_{c}}a_{T}^{-1}\left(T_{c}\left(t\right)\right)dt \end{array}$$

where $T_{g}$ is the glass transition temperature of PC, $t_{eff}^{c}$ is the effective time which accumulates during cooling, and $T_{c}\left(t\right)$ is the thermal history of the material experiences during processing. In essence the end-level of $t_{eff}^{c}$ is equal to the parameter $t_{a}$ [17,18,19]. The resulting yield stress can be subsequently determined by introducing $t_{eff}^{c}$ into Equation (1) [17,18,19]:

(5) $$\sigma_{y}=\sigma_{y,\;0}+c\cdot \log\left(t_{eff}^{c}\right)$$

The initial yield stress from processing can be calculated by Equation (5). The only variable is the effective time $t_{eff}^{c}$ determined by the thermal history of PC during processing. The thermal history during processing can be determined by means of finite element simulation of the injection molding process. To do this, the commercial injection molding simulation package MoldFlow is used. The obtained thermal history of each element is then exported to Equations (4) and (5) to calculate the initial yield stress from processing.

In our previous study [22], predictions of the yield stresses were compared with experimental data for PC tensile samples with different mold temperatures. The comparative results have proven the predictive capacity of the above model. However, it should be noted that a thickness-weighted average neglecting the inhomogeneous yield stresses distribution was used in [22] to enable the yields stress comparison and simplify the simulations. Obviously, the different thermal histories of the elements (for instance, the elements located near the surface have higher cooling rates) would lead to a heterogeneous yield stress distribution in the sample. Especially for a complex-shaped PC product, the difference of thermal history for the material elements at different locations is far larger compared with the simple-shaped products. In that case, the inhomogeneity of yield stress within the products is considerable and must be carefully described in the computation model, which is thus the primary objective of the present study.

## 3. Izod Impact Experiment

**Figure 1:**
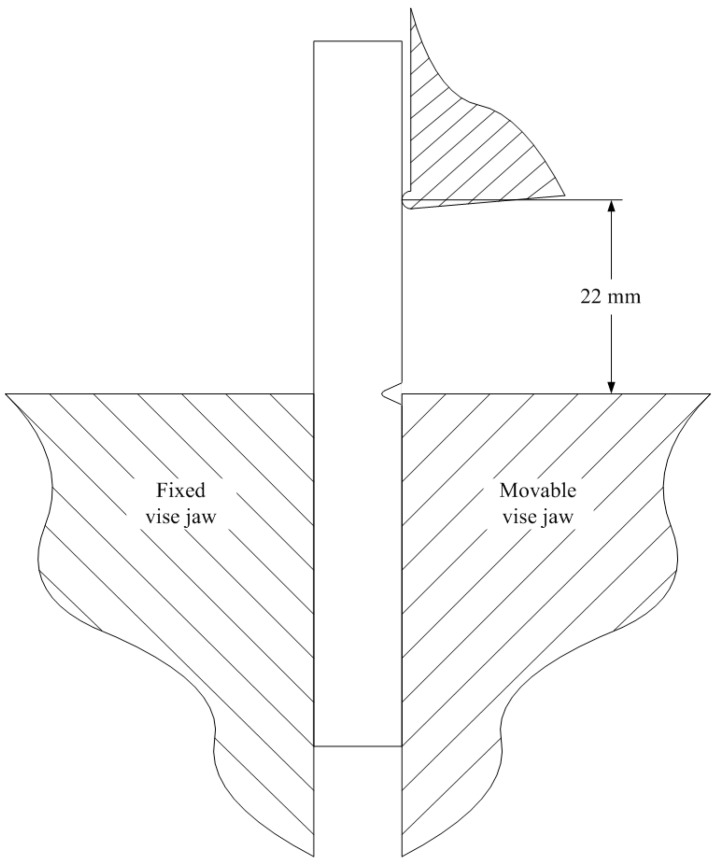
Illustration of the notched Izod impact test.

The notched Izod impact tests according to the ISO 180-2000 standard are carried out to analyze the impacting behaviors of PC samples with different thermal histories during injection molding. The notched Izod impact specimen, with dimension 80 × 10 × 3.2 mm^3^ and a 3 mm thickness and 30° V-shape notch in the center (see Figure 1), are directly injection molded. The melt temperature and injection velocity are set to 295 °C and 30 cm^3^/s. The packing pressure and cooling time are set to 65 MPa and 60 s. Three different mold temperatures 60, 80 and 120 °C, are used for the injection molding.

The impact test is conducted with a pendulum type impactor CEAST 9050. During the impact test, the bottom of the specimen is clamped as a cantilever beam. The upper part of the specimen is hit by the striking nose of the pendulum hammer with a controlled energy. As shown in Figure 1, the distance between the fixed support and the line of contact of the hammer’s striking nose is kept at 22 mm according to the standard. A calibrated hammer with a mass of 0.6 kg is used in the presented tests. The magnitudes of initial impact energy and velocity are varied by changing the initial angle of the hammer to fully fracture the PC specimens. Impact tests are performed respectively to determine the fracture energies of the specimens with different mold temperatures (60, 80, and 120 °C).

## 4. Integrative Simulation

**Figure 2:**
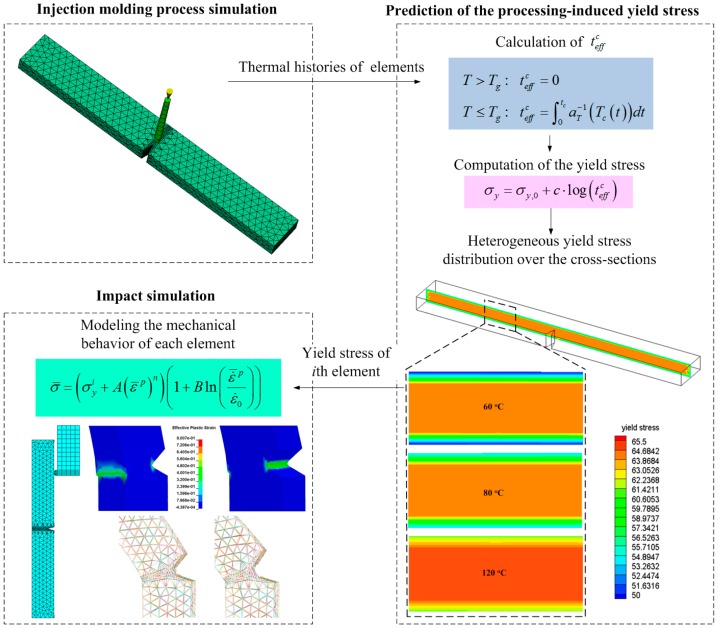
Illustration of the integrative simulation framework.

In this study, an integrative simulation framework is developed to incorporate the inhomogeneous yield stress distribution induced by injection molding into the mechanical performance simulations. The integrative simulation framework is schematically illustrated in Figure 2.

In the first step, the finite element model of specimen, developed in the commercial finite element software LSDYNA, is imported into MoldFlow for injection molding process simulation. Note that the injection molding meshes are exactly the same as the impact analysis meshes. In that case, the calculated yield stresses can be directly adopted in the impact analysis without interpolations.

In the second step, the thermal histories of all the elements during processing are obtained after the injection molding process simulation, which are further exported to Equation (5) to calculate the initial yield stresses from processing. Obviously, the different thermal histories of the elements leads to a heterogeneous yield stress distribution in the specimen.

In the last step, a modified strain rate dependent Johnson Cook model [22] combining the processing-induced yield stress is applied to model the mechanical behavior of each element in an impact simulation. The impact behaviors of specimens are finally analyzed in LSDYNA.

### 4.1. Prediction of Yield Stresses Based on the Injection Molding Simulation

**Figure 3:**
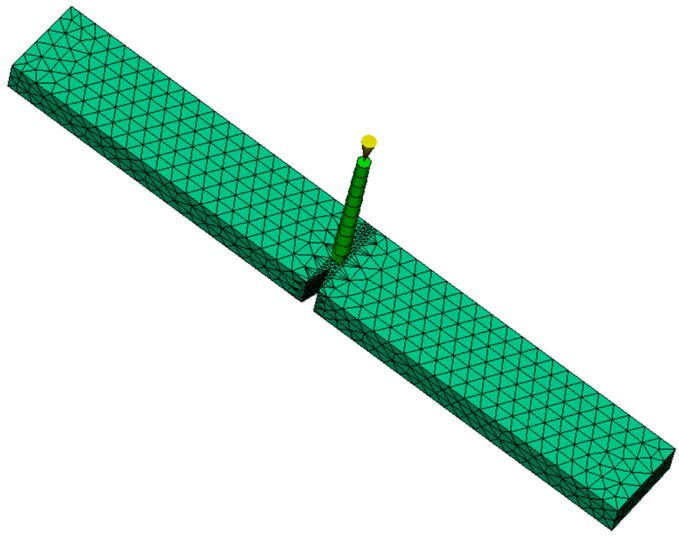
Finite element model of the specimen.

3D finite element model of the specimen used for the injection molding simulation is shown in Figure 3. Since the deformation is mostly concentrated in the vicinity of the notch, a finer mesh is chosen for this region. 36,368 elements with tetrahedral geometry are used to model the specimen. The process variables used for the injection molding simulation are identical to the ones in the experiment. The thermal history of each element is obtained for three different mold temperatures (60, 80 and 120 °C) and then exported to calculate the initial yield stresses.

**Figure 4:**
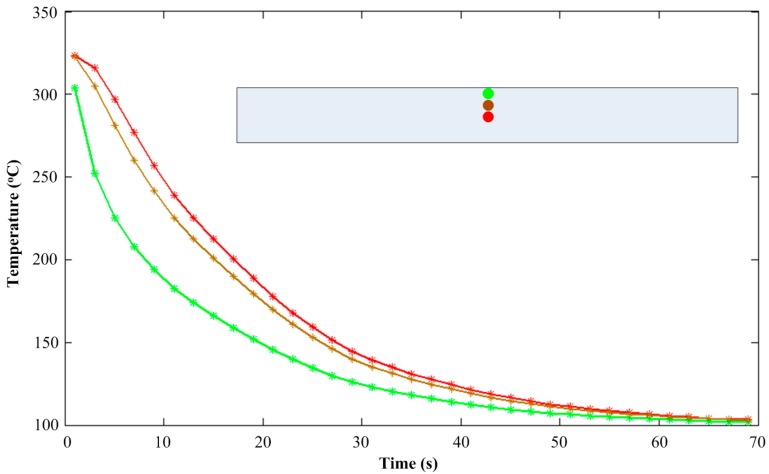
Cooling profiles of material elements at the center cross-section.

The cooling profiles of material elements at the center cross-section taken from the specimen for a mold temperature of 60 °C, as obtained from the numerical simulations, are shown in Figure 4. As expected, cooling is faster near the surface and slower at the center of the sample.

**Figure 5:**
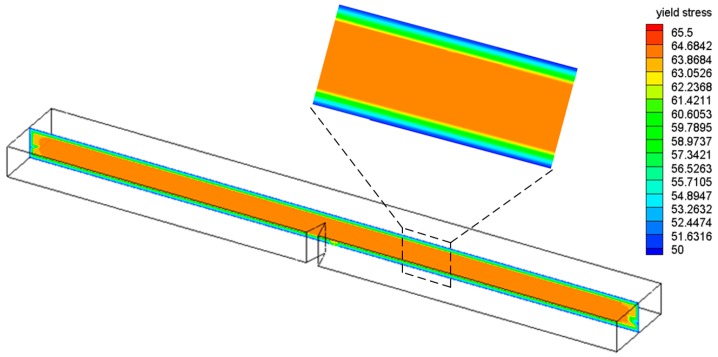
Distributions of yield stress within the center cross-section.

In Figure 5 the distributions of the yield stress are given for the center cross-section. A much lower yield stress can be found near the surface compared to that in the center, related to the higher cooling rates occurring there (see Figure 4).

**Figure 6:**
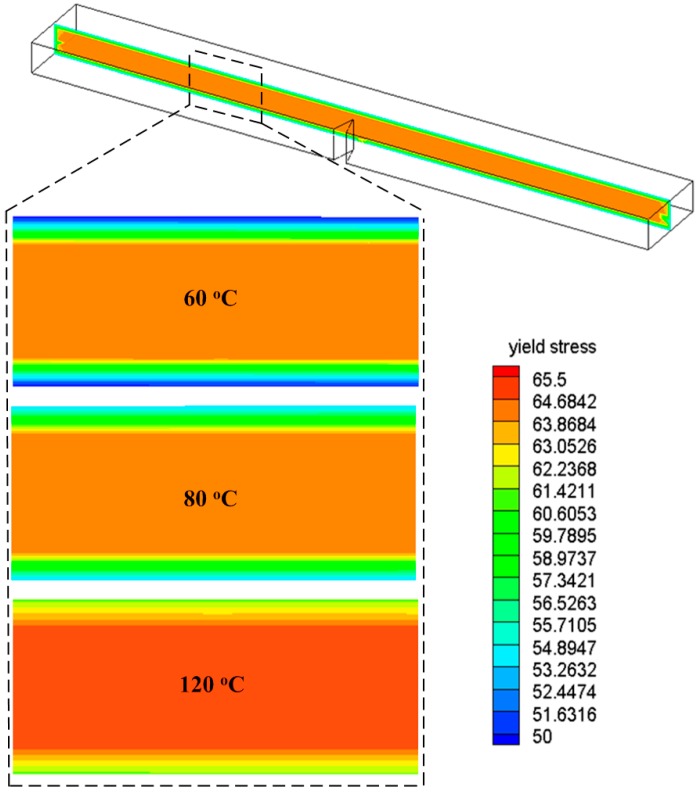
Yield stress distributions of the specimens with different mold temperatures.

In addition, Figure 6 lists the yield stress distributions at the center cross-section taken from the specimen for different mold temperatures. The yield stress distributions in the specimens with different mold temperatures are coincident. However, the value of yield stress is increased as the mold temperature increases.

### 4.2. Impact Simulation

In the impact simulation, a constitutive model proposed in our previous study [22] is used to model the mechanical behavior of each element. This constitutive model is developed by combining the processing-induced yield stress with a strain rate dependent Johnson Cook model:

(6) $$\bar{\sigma }=\left(\sigma_{y}^{i}+A{\left({\bar{\varepsilon }}^{p}\right)}^{n}\right)\left(1+B\ln\left(\frac{{\bar{\dot{\varepsilon }}}^{p}}{{\dot{\varepsilon }}_{0}}\right)\right)$$

where $\bar{\sigma }$ is the equivalent stress and ${\bar{\varepsilon }}^{p}$ is the equivalent plastic strain. The first term of the model describes the plastic behavior of the material. $\sigma_{y}^{i}$ is the yield stress of *i*th element. *A* and *n* are linked to the plastic flow. The second term reflects the strain rate sensitivity. ${\dot{\varepsilon }}_{0}$ is the reference strain rate and is set to 10^−2^ s^−1^ in this study. ${\bar{\dot{\varepsilon }}}^{p}$ is the equivalent plastic strain rate. The parameters *A* and *n* are identified on the basis of tests at the reference strain rate. The strain rate sensitivity parameter *B* is identified at the strain rates ranging from 10^−4^–10^−1^ s^−1^. The identified values of the model parameters are: *A* = 38 MPa, *n* = 2.815, and *C* = 0.079. The Young’s modulus and Poisson’s ratio of PC are 2271 MPa and 0.4, which are identified by the tensile tests.

It has been demonstrated that in a notched Izod impact test, the specimens with thin thickness (3.2 mm) undergo a ductile failure [22]. Thus a strain based failure criterion [22] is employed to model the ductile failure of the specimen. The failure indicator ψ is defined as:

(7) $$\psi =\frac{\sum \Delta {\bar{\varepsilon }}^{p}}{{\bar{\varepsilon }}_{\max}^{p}}$$

where ${\bar{\varepsilon }}_{\max}^{p}$ is a prescribed maximum equivalent plastic strain and is set to 1.0 according to the tensile test result. $\Delta {\bar{\varepsilon }}^{p}$ is the increment of equivalent plastic strain. An assumption is made that when the sum of the equivalent plastic strain increments at a material point is equal to or greater than the prescribed value of ${\bar{\varepsilon }}_{\max}^{p}$, *i.e.*, when ψ≥1, the material point fails and is permanently removed from future calculations.

**Figure 7:**
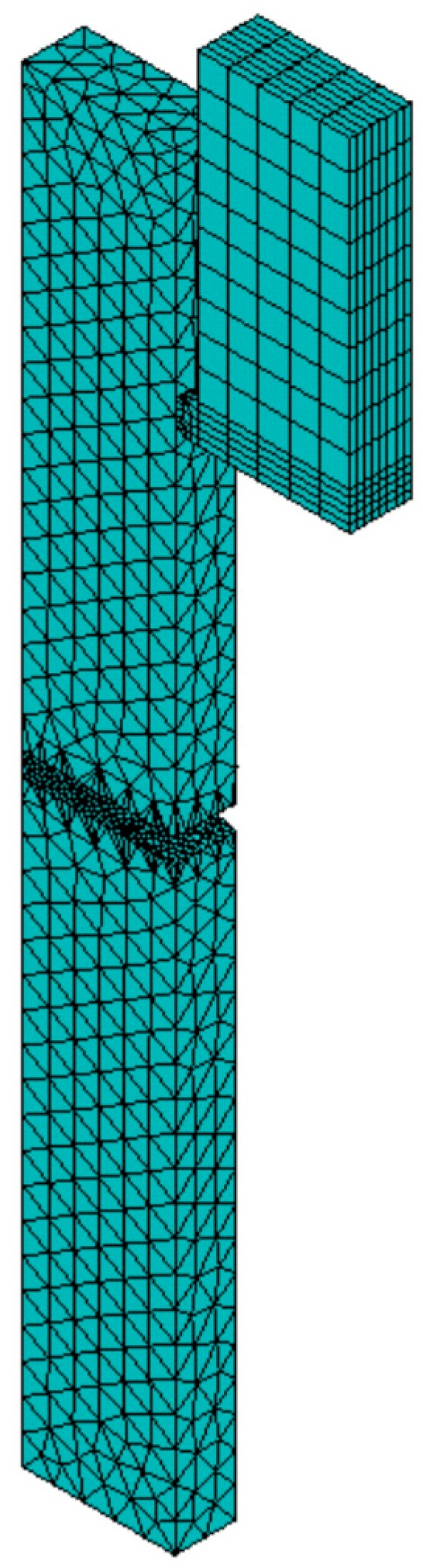
Finite element models of the specimen and pendulum hammer.

3D finite element models consisting of the specimen and pendulum hammer are displayed in Figure 7. Note that the impact analysis meshes are exactly the same as the injection molding meshes. Only a part of the hammer is modeled, but using a material density which to ensure the real energy and fully fracture the PC specimens. The hammer is assumed to be rigid with 200 GPa Young’s modulus and 0.3 Poisson’s ratio. A 3.5 m/s initial velocity in horizontal direction is applied to the hammer.

The edges of the Izod specimen are fully clamped to avoid any translational and rotational motion. All contact surfaces are assumed to be frictionless. Non-interpenetration of one material into the other is satisfied by using a penalty-based contact algorithm that considers the newly formed surfaces due to the deletion of failed elements.

### 4.3. Simulation Results and Comparison with Experimental Data

In this section, results of numerical simulations for the notched Izod impact behavior of PC specimens with different thermal histories are presented and compared with experimental data. For comparison, the impact simulations based on the thickness-weighted average neglecting the inhomogeneous yield stresses distribution are implemented as well.

**Figure 8:**
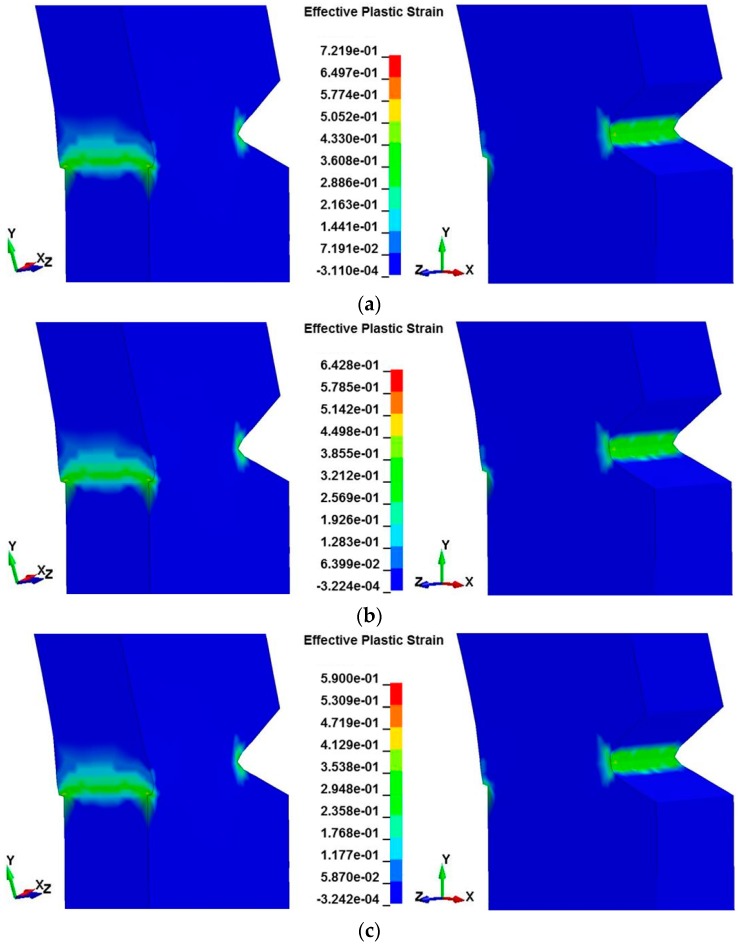
The distribution of plastic strain of the undamaged specimen with different model temperatures: (**a**) 60 °C; (**b**) 80 °C; (**c**) 120 °C.

The results of plastic strain distribution of the undamaged specimen with different model temperatures at 2.3 ms, obtained by the presented method, are displayed in Figure 8. Attention is focused on the distributions in the notch vicinity. The greatest straining occurs at the notch and there is also a high strain region opposite the notch where the bending strains are concentrated due to the nature of the constraint. The plastic strain is always higher for the specimen with a lower mold temperature. This is due to the process-induced yield behavior of PC. Since the yield stress of PC is increased as the mold temperature increases, earlier plastic deformation would be developed and higher levels of the plastic strain would be accumulated in the specimen with a lower mold temperature.

**Figure 9:**
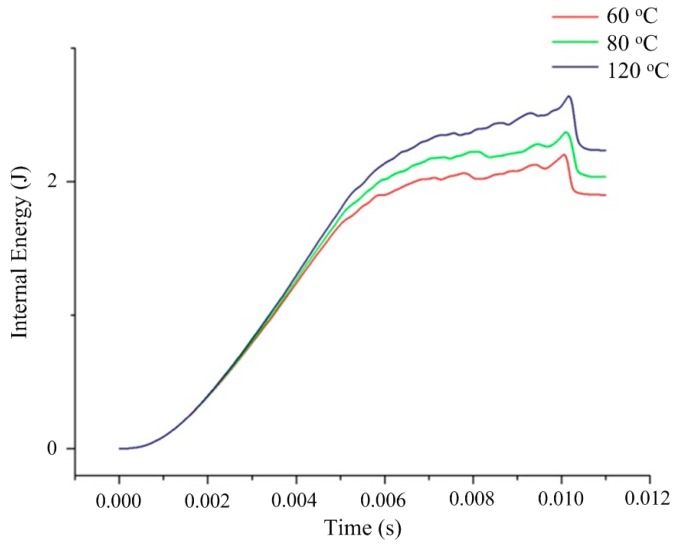
Internal energy variation of specimen with different mold temperatures during the impacting.

Figure 9 presents the variation of internal energy, *i.e.*, the total energy absorbed by the specimen with different mold temperatures during the impacting. Similar varying trends of the internal energies can be found for the impacting with different mold temperatures. The energies all increase gradually with the passing time and decrease suddenly in the last period, which means that all the specimens have been fully fractured. It is shown that the energy variation curves of the specimens with different mold temperatures are coalescing at up to nearly 3 ms. We can attribute this phenomenon to two aspects: (a) It is assumed that the PC specimens with different mold temperatures have the same elastic properties. Hence the variations of the energy with time are exactly the same before the yielding of material; (b) According to the prediction results of yield stresses, the differences among yield stresses of the specimens are not significantly large to observably affect the energy curves in the initial stage of the post-yielding deformation. Thus we cannot expect a dramatic difference among the energies. However, distinct separations of the energy curves are found after the initial stage, especially in the fracture process.

**Figure 10:**
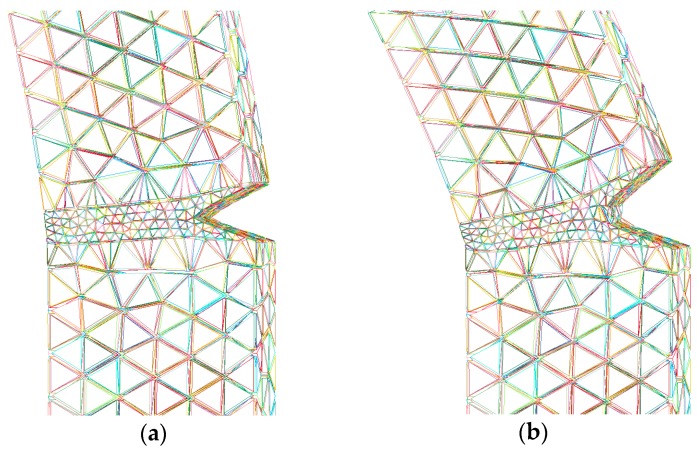

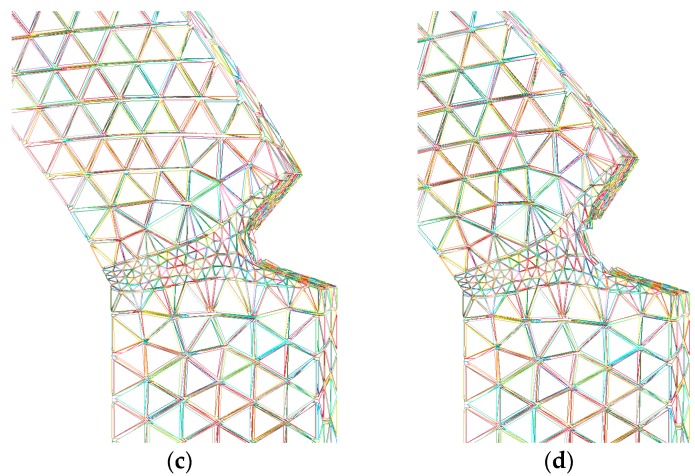
Fracture process of the specimen with 60 °C mold temperature: (**a**) 1.54 ms; (**b**) 4 ms; (**c**) 5.8 ms; (**d**) 7.8 ms.

A detailed fracture process of the specimen with 60 °C mold temperature is displayed in Figure 10. The fracture energy can be calculated by dividing the total energy absorbed by the specimen at the cross-sectional area at the notch. The fracture energies of numerical simulations and experimental tests for the specimens with different mold temperatures are listed in Table 1. The predicted fracture energies of the PC specimens with different mold temperatures are shown to be in good agreement with the experimental data. Both experimental and numerical results clearly indicate that the fracture energy increases as the mold temperature increases. Since the yield stress of PC is increased as the mold temperature increases, more energy of the striker would be consumed for plastically deforming and fracturing the PC specimen with a higher mold temperature. The numerical results based on the average method are listed in Table 1 as well. It is clearly shown that an improved agreement between experimental results and simulations is achieved considering the inhomogeneity of yield stress. The fracture energy predicted by the average method (neglecting the inhomogeneity of yield stress) is always lower than the presented method (considering the inhomogeneity of yield stress). We can attribute this phenomenon to the reduction of yield stresses in the core of the specimen caused by the average. As shown in Figure 6, the yield stress in the core is far bigger than the yield stress in the surface (the difference is about 15 MPa). Therefore, the average of yield stresses from surface to core leads to a decrease of the yield stress in the core, which induces the earlier plastic deformation and lower fracture energy.

## 5. Conclusions

In this paper, an integrative simulation framework is developed to incorporate the inhomogeneous yield stress distribution induced by injection molding into the mechanical performance simulations of PC products. A modeling approach developed by Govaert, *et al.* [17,18,19] for predicting the yield stress from the temperature history experienced during injection molding process is used in this study. Experimental test and finite element simulation for notched Izod impact tests are performed to analyze the impact behaviors of PC samples with different thermal histories. Compared to our previous study [22], the inhomogeneous yield stress distribution along the injection molded specimen is calculated and used for the notched Izod impact simulations, instead of an average value for the whole specimen.

The fracture energies of the PC specimens obtained by numerical simulation and experimental tests coincide well. Both experimental and numerical results indicate that the fracture energy increases as the mold temperature increases. We can attribute this phenomenon to the process-induced yield behavior of PC. Since the yield stress of PC is increased as the mold temperature increases, more energy of the striker would be consumed for plastically deforming and fracturing the PC specimen with a higher mold temperature.

## Figures and Tables

**Table 1:** Fracture energies of numerical simulations and experimental tests.

Mold temperature (°C)	Method	60	80	120
Fracture energy (kJ/m^2^)	Presented method	79.81	84.66	88.30
	Average method	77.53	82.38	86.94
	Experimental	85.74	90.18	95.63
